# Characterization of Feedback Neurons in the High-Level Visual Cortical Areas That Project Directly to the Primary Visual Cortex in the Cat

**DOI:** 10.3389/fnana.2020.616465

**Published:** 2021-01-08

**Authors:** Huijun Pan, Shen Zhang, Deng Pan, Zheng Ye, Hao Yu, Jian Ding, Qin Wang, Qingyan Sun, Tianmiao Hua

**Affiliations:** College of Life Sciences, Anhui Normal University, Wuhu, China

**Keywords:** top-down influence, feedback neurons, primary visual cortex, cat, high-level visual cortex

## Abstract

Previous studies indicate that top-down influence plays a critical role in visual information processing and perceptual detection. However, the substrate that carries top-down influence remains poorly understood. Using a combined technique of retrograde neuronal tracing and immunofluorescent double labeling, we characterized the distribution and cell type of feedback neurons in cat’s high-level visual cortical areas that send direct connections to the primary visual cortex (V1: area 17). Our results showed: (1) the high-level visual cortex of area 21a at the ventral stream and PMLS area at the dorsal stream have a similar proportion of feedback neurons back projecting to the V1 area, (2) the distribution of feedback neurons in the higher-order visual area 21a and PMLS was significantly denser than in the intermediate visual cortex of area 19 and 18, (3) feedback neurons in all observed high-level visual cortex were found in layer II–III, IV, V, and VI, with a higher proportion in layer II–III, V, and VI than in layer IV, and (4) most feedback neurons were CaMKII-positive excitatory neurons, and few of them were identified as inhibitory GABAergic neurons. These results may argue against the segregation of ventral and dorsal streams during visual information processing, and support “reverse hierarchy theory” or interactive model proposing that recurrent connections between V1 and higher-order visual areas constitute the functional circuits that mediate visual perception. Also, the corticocortical feedback neurons from high-level visual cortical areas to the V1 area are mostly excitatory in nature.

## Introduction

It is widely assumed for a long time that visual perception is formed step by step in a feedforward mode from the retina to LGN, then to the primary visual cortex (V1), and finally to higher-order visual cortical areas (Hubel and Wiesel, 1968; Hirsch, 2003; Herzog and Clarke, 2014). Different types of visual information are processed through segregated pathways of ventral and dorsal streams in the visual cortex of primates and cats (Tong et al., 2011; Sheth and Young, 2016). Generally, the ventral stream, from V1 to V3 and then to V4, processes information of object form and identity, whereas the dorsal stream, from V1 to V2 and then to V5 (MT), is responsible for the processing of object location and movement (Sheth and Young, 2016). Anatomical and physiological evidence has shown that the cortical areas 17, 18, and 19 of the cat can be equated with macaque areas V1, V2, and V3, respectively, and the area 21a and the posterior medial bank of the lateral suprasylvian sulcus (PMLS) can be equated with macaque areas V4 and V5, respectively (Payne, 1993; Dreher et al., 1996a; Price et al., 2006; Shen et al., 2006; Tong et al., 2011). Therefore, the visual system of a cat may have a ventral-dorsal information processing manner similar to that of primate (Dreher et al., 1996b; Wang et al., 2007; Tong et al., 2011), but supporting evidence is quite limited so far (Connolly et al., 2012).

On the other hand, although the feedforward visual signal encoding along the hierarchical visual pathways is fundamental, an increasing body of evidence indicates that top-down influence from higher-level cortical areas to the V1 area plays a critical role in information processing and visual perception (Galuske et al., 2002; Lee, 2002; Ro et al., 2003; Gazzaley et al., 2005; Fenske et al., 2006; Schwabe et al., 2006; Gilbert and Sigman, 2007; Rolls, 2008; Bardy et al., 2009; Chalk et al., 2010; McMains and Kastner, 2011; Al-Aidroos et al., 2012; Nassi et al., 2013; Moldakarimov et al., 2014; Zhang et al., 2014; Kamiyama et al., 2016; Liang et al., 2017; Nurminen et al., 2018; Keller et al., 2020). However, the characteristics of feedback influence on the responses of V1 neurons remains in debate (Han and VanRullen, 2016). Some authors propose that top-down influence produces excitatory feedback inputs and facilitate neuronal response in the V1 area (Wang et al., 2000, 2007, 2010; Huang et al., 2004; Liang et al., 2007; Tong et al., 2011; Chen et al., 2014; Zhang et al., 2014; van Loon et al., 2015; Kok et al., 2016; Pafundo et al., 2016; Yang X. et al., 2016; Baumgartner et al., 2018; Huh et al., 2018), whereas others suggest that top-down influence exert suppressive impacts on neurons in the low-level visual areas (Roland et al., 2006; Chalk et al., 2010; Nassi et al., 2013, 2014; Klein et al., 2014; Hishida et al., 2019; Maniglia et al., 2019). Still, others report bidirectional top-down effects of both suppression and enhancement (Gazzaley et al., 2005; Johnson and Johnson, 2009; Cox et al., 2019). Furthermore, though the top-down influence of different higher visual cortical regions is widely reported, their relative contributions to the information encoding of V1 neurons are largely unclear (Huang et al., 2007; Wang et al., 2007; Huh et al., 2018).

To understand the above-mentioned issues, a critical step is to examine the corticocortical connection substrates that carry top-down influence on the low-level cortical areas. Although some authors have taken efforts to define the feedback projections using retrograde and anterograde tracing techniques (Olson and Lawler, 1987; Dreher et al., 1996a; Fitzgibbon et al., 1999; Han et al., 2008; Connolly et al., 2012; Yang X. et al., 2016), information about the distribution and cell types of feedback neurons in different high-level cortical areas are quite limited. Using a combined technique of retrograde neuronal tracing and immunofluorescent double labeling, this study compared the proportion and cell type of neurons in cat’s different higher-level visual cortical areas that send direct feedback projections to the V1 area, trying to expand our understanding of the characteristics and mechanisms of top-down influence.

## Materials and Methods

### Animals

Five healthy young adult cats (female, 1–3 years old, bodyweight 2.5–3.4 kg) were used in this study. All cats had normal vision with no retinal and eye disease. All animal treatments and experimental procedures were strictly following the National Institutes of Health Guide for the Care and Use of Laboratory Animals and were approved by the Academic and Ethics Committee of Anhui Normal University.

### Animal Preparation and Injection of Retrograde Tracer

Animal anesthesia and physiological maintaining were performed as previously described (Hua et al., 2010; Meng et al., 2013; Yang J. et al., 2016; Zhao et al., 2020). Briefly, the cat was first anesthetized with ketamine HCl (40 mg/kg, i.m.) and xylazine (2 mg/kg, i.m.). Noninvasive intubation of tracheal and intravenous cannulae was performed under sterile preparation. After the cat was fixed in a stereotaxic apparatus, glucose (5%)-saline (0.9%) solution containing urethane (40 mg/kg body weight) was infused intravenously to maintain necessary anesthesia. Artificial respiration was performed and expired pCO2 was kept at approximately 3.8%. Heart rate (approximately 180–220 pulses/min) and electrocardiogram were monitored during the experiment to evaluate the state of anesthesia and ensure the animals were not responding to pain. The body temperature (38°C) was maintained using a heating blanket.

Microinjection of neuronal tracers was delivered *via* a pulled glass micropipette (tip diameter 10–15 μm) attached to a 2-μl Hamilton syringe. We selected red Retrobeads (#78R170, Lumafluor Inc., Shanghai, China), a fluorescent dye, as the retrograde neuronal tracer as previously reported (Zhang et al., 2014). The injection of red Retrobeads was performed in the V1 area (Horsley-Clarke coordinates: P0-P8/L0-L4) of the left hemisphere after a craniotomy on the skull. We selected six injection sites in the V1 area (P1/L1.5, P2/L2, P3/L2.5, P4/L3, P5/L3.5, P6/L3.5), which corresponded to the retinotopic coordinates within approximately 0–20° from the vertical and horizontal meridian according to previous studies (Tusa et al., 1978, 1979; Connolly et al., 2012; see Supplementary Information: Supplementary Figure 1). In each injection site, a total of 1 μl red Retrobeads was delivered slowly and separately at different cortical depth (2,000–200 μm, the release of 0.1 μl at an interval of 200 μm) from the cortical surface. At the end of the injection, the exposed cortical area was covered with absorbable gel foam, and the opening was closed with a piece of the repaired skull using tissue adhesive and dental cement. After the incision was sutured, the anesthesia supply was terminated. The animal was moved to the rearing room after it recovered to a normal physiological state. Full care was given to the animal in the following 2 weeks. On the first 3–4 days after surgery, the animal was given a daily dose injection (1 ml) of antibiotic penicillin (800,000 units) to protect against infection.

### Brain Tissue Sectioning and Immunofluorescent Double Labeling

Two weeks after tracer injection, the cat was deeply anesthetized with ketamine HCl (80 mg/kg, i.m.) and then transcardially perfused with 0.9% saline followed by 2% paraformaldehyde in 0.1 M phosphate-buffered saline (PBS). The brain tissue on the left hemisphere was removed and post-fixed overnight in 2% paraformaldehyde at 4°C. On the next day, the cerebral cortex containing visual cortical areas 17, 18, 19, 21a, and PMLS was dissected and cryoprotected by sequential incubation in 10% (2 h), 20% (2 h), and 30% (overnight) sucrose until tissue sinking. Then, the brain tissue was embedded in OCT compound (Tissue-Tek, 4583, Sakura Finetek Inc., Torrance, CA, USA), and coronal sections were cut at a thickness of 40 μm using a Leica cryostat (Leica Biosystems Inc., Buffalo Grove, IL, USA). Serial frozen sections were collected in order, placed in wells filled with cryoprotectant solution (ethylene glycol-based; 30% ethylene glycol, 30% sucrose, 1% PVP-40, in 0.1 M Phosphate buffer pH 7.4) and temporarily stored at −20°C for subsequent observation and immunofluorescent labeling.

We visualized respectively the total cortical neurons (NeuN-labeled neurons), CaMKII-positive excitatory neurons, and GABAergic inhibitory neurons on adjacent free-floating sections using the fluorescent double-labeling technique. The primary antibodies used in this study included rabbit anti-NeuN (1:1,000, ab177487, Abcam, Shanghai, China), rabbit anti-CaMKII (1:120, ab134041, Abcam, Shanghai, China), and rabbit anti-GABA (1:200, A2052, Sigma, Shanghai, China). After incubation overnight at 4°C with primary antibodies, the sections were washed in PBS for three times, and then incubated with the secondary antibody (goat anti-Rabbit IgG H&L, Alexa Fluor 488, 1:1,000, ab150077; Abcam) diluted in QuickBlock Secondary Antibody Dilution Buffer (P0265; Beyotime) for 2 h at room temperature. After secondary antibody incubation and several washes in PBS, sections were mounted on clean glass slides with glycerol and sealed with nail polish. Control sections were labeled simultaneously using the same procedure as described above, with the exception that the primary antibody was substituted with QuickBlock Primary Antibody Dilution Buffer.

### Image Acquisition and Statistical Analysis

Images were taken with a confocal laser scanning microscope (FV1000, Olympus) using a 20× or 60× objective as described in our previous studies (Ding et al., 2017, 2018). Automated sequential acquisition of multiple channels was used. The frame size was 1,024 × 1,024 pixels or 512 × 512 pixels. For each image, 10 confocal planes were *Z*-stacked with a step of 0.56 μm. Stacks of images were merged into a maximum intensity projection and saved as a tiff file.

**Figure 1:**
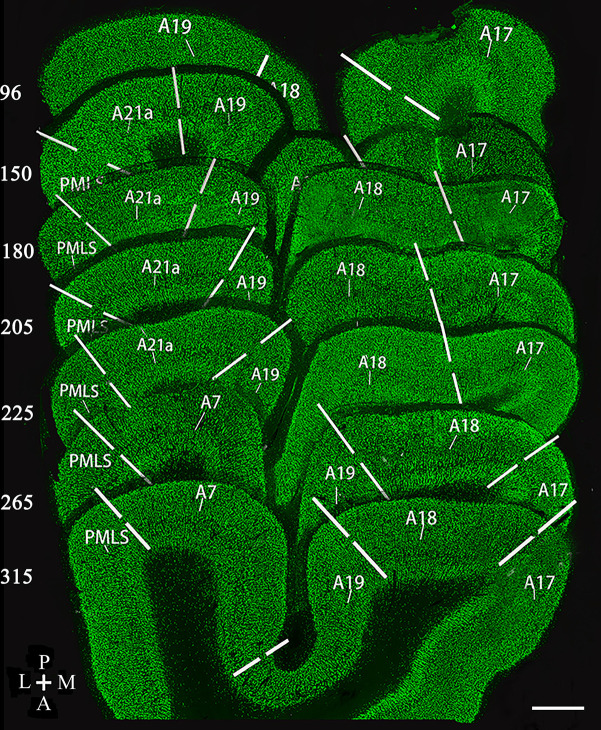
Reconstruction of coronal section samples showing estimates of the border (solid lines) between visual cortical area 17 (A17), 18 (A18), 19 (A19), 21a (A21a), PMLS, and 7 (A7) according to previous studies (Payne, 1993; Dreher et al., 1996b; Rushmore and Payne, 2004; Huang et al., 2006; Connolly et al., 2012; Stolzberg et al., 2017). The number on the left of each section indicates the serial section number counted along with posterior (P)-to-anterior (A) direction, which corresponds approximately to the Horsley–Clarke coordinates at P8, P4, P3, P2, P1, A2, and A4. The section thickness is 40 μm. The scale bar (in the lower right corner) equals to 1,000 μm.

Ten randomly sampled slice triplets, including adjacent NeuN-, CaMKII- and GABA-labeled slices, in each cortical area from each animal, were used for data analysis. The visual cortical areas, including area 17 (A17), area 18 (A18), area 19 (A19), area 21a (A21a), and area PMLS, were located according to Horsley-Clarke coordinates of cat brain (Payne, 1993; Dreher et al., 1996b; Rushmore and Payne, 2004; Huang et al., 2006; Connolly et al., 2012; Stolzberg et al., 2017) after reconstruction with serial coronal sections labeled with NeuN (Figure 1). Cell counting was carried out at the central location of each visual cortical area and performed using Image-Pro Plus 6.0 software (MediaCybernetics, Bethesda, MD, USA) by experimenters who were blinded to the cortical areas and animals from which the images were obtained.

**Figure 2:**
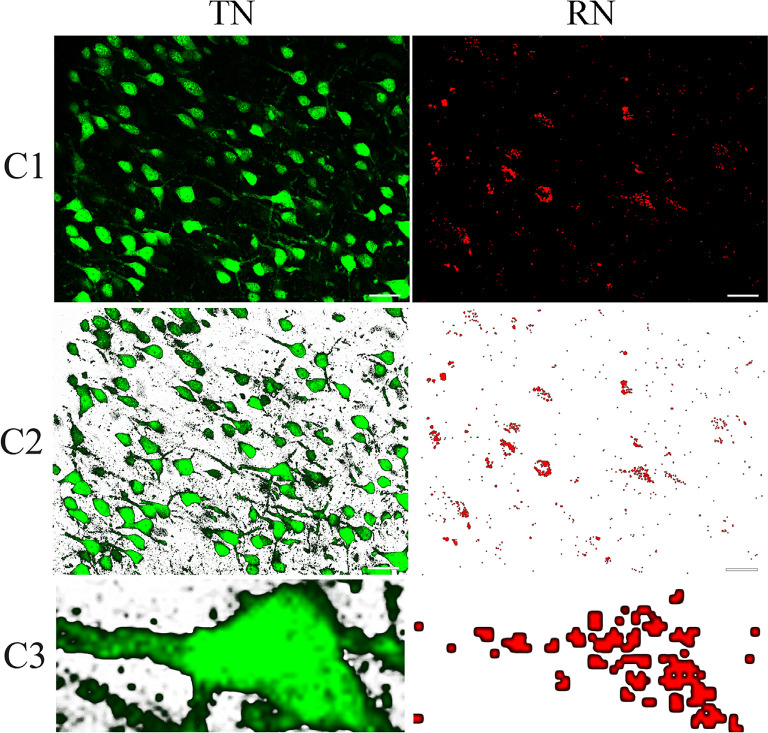
Sample contours showing the method for computing the overlapping goodness between red Retrobeads-labeled neurons (right column) and NeuN-labeled neurons (left column). (C1) shows a typical NeuN/Retrobeads double-positive neuron (indicated by dashed line boxes) in the corresponding area of images from cortical layer II/III in the area 21a. (C2) shows the NeuN/Retrobeads double-positive neuron (dashed line boxes) in the corresponding area of images with the background-subtracted using tools of Photoshop CS3. (C3) Shows an extracted contour of NeuN-labeled neuron (NN) and the corresponding Retrobeads-labeled neuron (RN) from the images. The paired contours of neurons were loaded into MATLAB 2014a for calculating their shape overlapping degree (%) using Hamming distance computing program (School: Brandeis University. Professor: Praveen Chaturvedi compare shape: amount of overlap with hamming distance. See Supplementary File 1: compare shape.m). The scale bar equals to 30 μm.

To determine if a red Retrobeads-traced neuron (RN) had a good overlap with a NeuN-positive neuron (NN) or CaMKII-positive excitatory neuron (CN) or GABA-positive inhibitory neuron (GN), we computed the similarity degree between the RN and the corresponding NN or CN or GN in the paired contours of neurons (Figure 2). Briefly, after extracting the contours of corresponding neurons using Image-Pro Plus software, the paired contours were loaded into Matlab 2014a, and their shape overlapping degree (%) was calculated using Hamming distance computing program (Brandeis University, Professor Praveen Chaturvedi; compare shape: amount of overlap with hamming distance. See Supplementary File 1: compare shape.m). An RN had a contour overlapping degree of ≥75% with the corresponding NN, CN, and GN was defined as a NeuN/Retrobeads double-positive neuron (NRN), CaMKII/Retrobeads double-positive neuron (CRN) and GABA/Retrobeads double-positive neuron (GRN), respectively.

The number of different types of neurons, including NN, NRN, CRN, and GRN neurons, was counted in the corresponding area of interest (AOI, 100 × 100 μm) at each cortical layer (layers I, II–III, IV, V, and VI) in stacked images from each sampled slice using “Image-Pro Plus” AOI duplicate function. The cortical layers were identified according to the adjacent NeuN-labelled section. The mean density of NN, NRN, CRN, and GRN in each cortical layer were calculated based on the cell count across multiple AOIs.

The mean value in each cortical area of each animal was expressed as the mean ± standard deviation. Comparisons between different cortical layers and between different cortical areas were performed with ANOVA or nonparametric tests. The difference with *p* < 0.05 was considered significant.

## Results

### Neurons Traced by Red Retrobeads in the Different High-Level Visual Cortex

**Figure 3:**
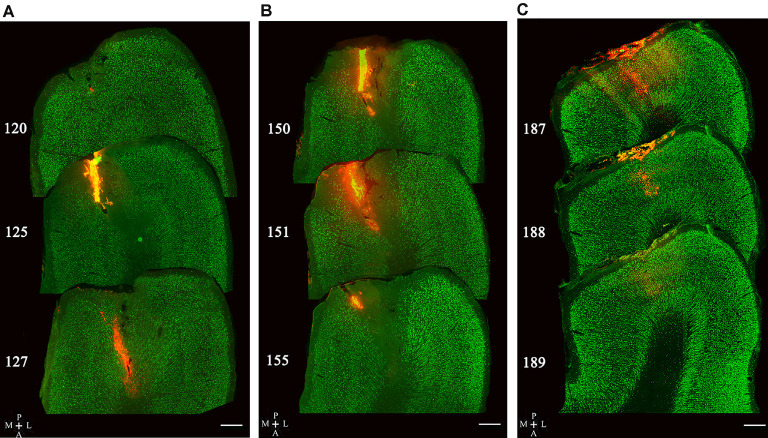
Image samples showing Retrobeads (red fluorescence) injection site across multiple sections with double-labeling of NeuN (green fluorescence). Superposed images in **(A–C)** display three different injection sites in the V1 area (area 17). The number on the left of each section indicates the serial section number counted along the posterior-to-anterior direction. The scale bar equals to 500 μm.

The injection sites of red Retrobeads in the V1 area of five cats were examined in consecutive sections. The tracer delivery in three of five cats was successful with all injection locations within the gray matter of the V1 area, and no visible neuronal damage was observed around injection sites (Figures 3A–C). Two cats showed some injection locations deep into the white matter of the V1 area and thus were not used for statistical counting of traced neurons.

**Figure 4:**
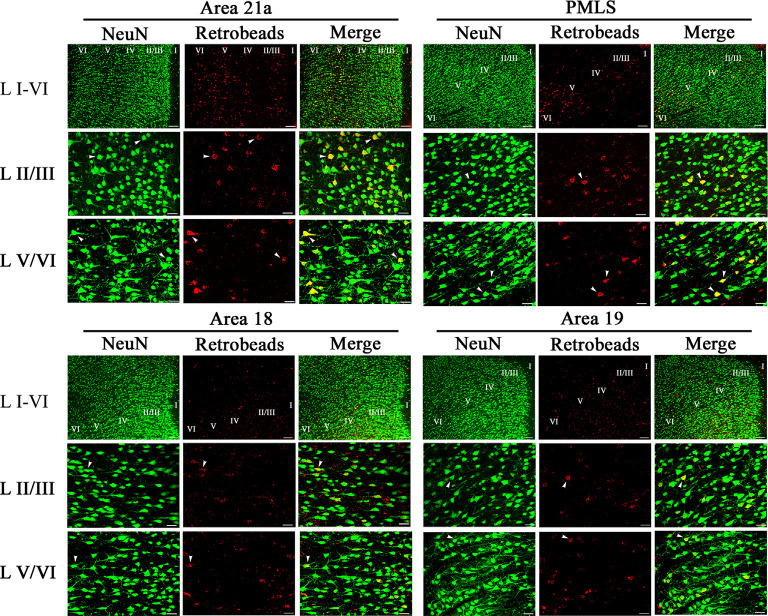
Samples of images showing the distribution of NeuN-labeled total neurons (NN), red Retrobeads-labeled neurons (RN), and NeuN/Retrobeads double-positive neurons (NRN) at different cortical layers in the cortical area 21a (upper left square), PMLS (upper right square), 18 (lower left square) and 19 (lower right square). The row L I–VI shows the distribution of NNs, RNs, and NRNs in the cortical layer 1–6 at low magnification power, and the scale bar equals to 120 μm. The row L II/III and L V/VI show NNs, RNs, and NRNs in the cortical layer 2/3 and 5/6 at high magnification power, with the scale bar of 30 μm. The letter I, II/III, IV, V, and VI indicate the cortical layers 1, 2/3, 4, 5, and 6, respectively. Arrows indicate the typical neurons of NN, RN, and NRN.

NeuN-labeled neurons (NNs) with cell bodies traced by red Retrobeads were found in all studied high-level visual cortex (Figure 4). A Retrobeads-traced neuron (RN) with ≥75% overlap with a NN was counted as a feedback or NRN neuron. Observation on sampled sections found that NRN neurons in A21a and PMLS area were denser than that in A18 and A19 (Figure 4), and most NRNs distributed at layer 2–3 (II–III), layer 5 (V), and layer 6 (VI), a small number of NRNs presented at layer 4 (IV), and no NRN was identified in layer 1 (I; Figure 4). Therefore, we statistically compared the proportion of NRN to NN at layers II–III, IV, V, and VI between different high-level visual cortical areas.

**Figure 5:**
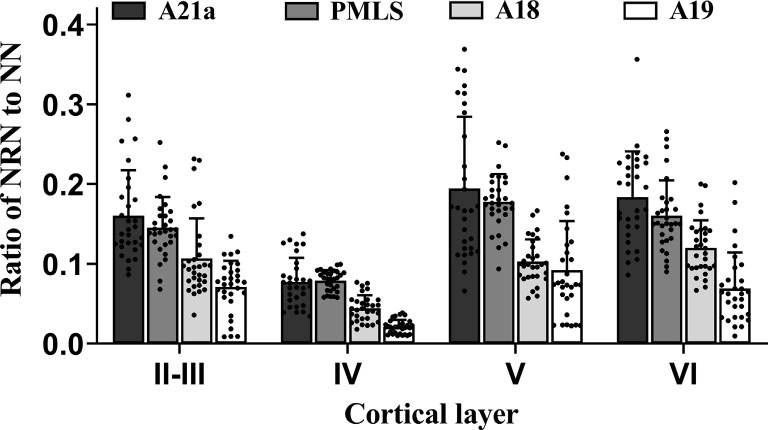
Histograms with error bars (SDs) represent the mean proportion of NeuN/Retrobeads double-labeled neurons (NRNs) to NeuN-labeled total neurons (NNs) at cortical layer II–III, IV, V, and VI in cortical area 21a (A21a), PMLS, 18 (A18), and 19 (A19). Solid dots on each histogram show individual data of count from 30 sections (10 sections/cat).

Two-way ANOVA indicated that the mean proportion of NRN to NN showed significant variation between different high-level visual cortical areas (main effect of the area: *F*_(3,464)_ = 105.239, *p* < 0.0001), and between different cortical layers (effect of layer: *F*_(3,464)_ = 91.904, *p* < 0.0001; Figure 5); there was a significant interaction between cortical area and cortical layer (interaction of area × layer: *F*_(9,464)_ = 2.709, *p* = 0.004). Further *Post hoc* pairwise comparisons between cortical areas indicated that the mean proportion of NRN to NN across all cortical layers in A21a had no significant difference from that in the PMLS area (*p* = 0.101), whereas that in both A21a and PMLS area was significantly higher than that in A18 and A19 (all *p* < 0.0001), and that in A18 was significantly larger than in A19 (*p* < 0.0001). *Post hoc* pairwise comparisons between cortical layers showed that the mean value of NRN/NN across all cortical areas in layer VI was not significantly different from that in layer II–III (*p* = 0.145) and layer V (*p* = 0.453), whereas that in layer IV was significantly lower than in layer II–III, V and VI (all *p* < 0.0001), and that in layer II–III was smaller than in layer V (*p* = 0.002).

All analysis above indicated that the feedback neurons in high-level visual cortical areas had a denser distribution in cortical layer II–III (0.12 ± 0.06), V (0.14 ± 0.07), and VI (0.13 ± 0.06) than in layer IV (0.06 ± 0.03), and the proportion of feedback neurons was higher in A21a (0.15 ± 0.08) and PMLS area (0.14 ± 0.05) than in A18 (0.09 ± 0.04) and A19 (0.06 ± 0.05).

### Identification of the Cell Type of Feedback Neurons

To evaluate the proportion of excitatory and inhibitory cell types of feedback neurons in these high-level visual areas, we respectively measured and compared the mean ratio of CaMKII/Retrobeads double-positive neurons (CRNs) and GABA/Retrobeads double-positive neurons (GRNs) to NNRs at each cortical layer of different cortical areas in neighboring sections.

#### CaMKII-Positive Feedback Neurons

**Figure 6:**
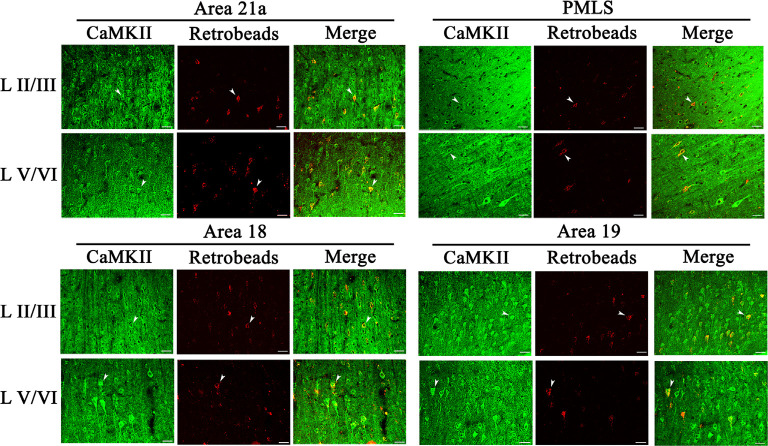
Samples of images showing the distribution of CaMKII-positive neurons (CN), red Retrobeads-labeled neurons (RN), and CaMKII/Retrobeads double-positive neurons (CRN) at different cortical layers in the cortical area 21a (upper left square), PMLS (upper right square), 18 (lower left square), and 19 (lower right square). The row L II/III and L V/VI respectively show CNs, RNs, and CRNs in the cortical layer 2/3 and 5/6 at a high magnification power. Arrows indicate the typical neurons of CN, RN, and CRN. The scale bar equals to 30 μm.

The excitatory CaMKII-positive neurons (CN) distributed widely across all cortical layers in each high-level visual cortex, and pyramidal cells in layers III and V showed a stronger immunoreaction than in layers IV and VI (Figure 6). Observation in all sampled sections found that red Retrobeads-traced neurons (RNs) in A21a and PMLS areas had more overlapping with CNs compared with that in A18 and A19, and the CaMKII/Retrobeads double-positive neurons (CRNs) were denser in cortical layer II–III, V, and VI than in layer IV (Figure 6).

**Figure 7:**
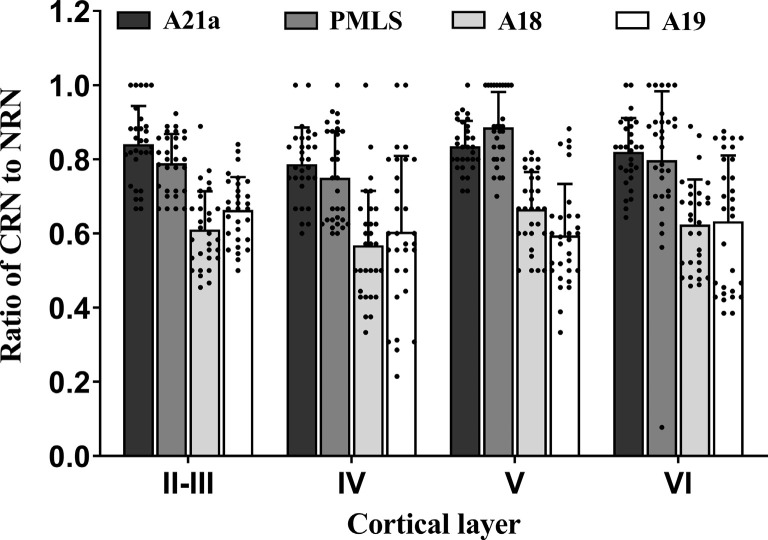
Histograms with error bars (SDs) represent the mean proportion of CaMKII/Retrobeads double-labeled neurons (CRNs) to NeuN/Retrobeads double-labeled neurons (NRNs) at cortical layer II–III, IV, V, and VI in the cortical area 21a (A21a), PMLS, 18 (A18), and 19 (A19). Solid dots within each histogram show individual data of counts from 30 neighboring sections (10 sections/cat).

Two-way ANOVA indicated that the proportion of CRN neurons to NRNs showed a significant variation among different high-level visual cortical areas (main effect of the area: *F*_(3,464)_ = 93.38, *p* < 0.0001), and between different cortical layers (effect of layer: *F*_(3,464)_ = 6.096, *p* < 0.0001; Figure 7); there was a significant interaction between cortical areas and cortical layers (interaction of area × layer: *F*_(9,464)_ = 1.96, *p* = 0.042). Further *Post hoc* pairwise tests between cortical areas showed that the mean proportion of CRNs to NRNs across all cortical layers had no significant difference between in A21a and PMLS (*p* = 0.80) as well as between A18 and A19 (*p* = 0.977), whereas the mean ratio of CRNs to NRNs in both A21a or PMLS area was significantly higher than in A18 and A19 (all *p* < 0.0001). *Post hoc* pairwise tests between different cortical layers displayed that the mean value of CRNs/NRNs across all cortical areas exhibited no significant difference among cortical layer II–III, V, and VI (layer II–III vs. V: *p* = 0.24; layer II–III vs. VI: *p* = 0.651; layer V vs. VI: *p* = 0.104), whereas the mean value of CRNs/NRNs in layer IV was significantly smaller than in layer II–III (*p* = 0.003), V (*p* < 0.0001) and VI (*p* = 0.012).

All comparisons displayed above indicated that the mean proportion of excitatory feedback neurons was higher in A21a (0.82 ± 0.09) and PMLS area (0.80 ± 0.13) than in A18 (0.61 ± 0.12) and A19 (0.62 ± 0.15) and the excitatory feedback neurons had a denser distribution at cortical layer II–III (0.73 ± 0.13), V (0.75 ± 0.15), and VI (0.72 ± 0.17) than at layer IV (0.67 ± 0.17).

### GABA-Positive Feedback Neurons

**Figure 8:**
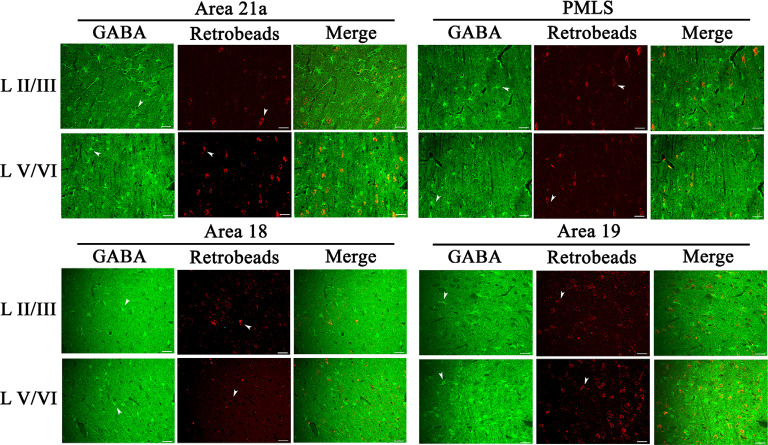
Samples of images showing the distribution of GABA-positive neurons (GN), red Retrobeads-labeled neurons (RN), and GABA/Retrobeads double-positive neurons (GRN) at different cortical layers in the cortical area 21a (upper left square), PMLS (upper right square), 18 (lower left square), and 19 (lower right square). The row L II/III and L V/VI respectively show GNs, RNs, and GRNs in the cortical layer 2/3 and 5/6. Arrows indicate the typical neurons of GN and RN. The scale bar equals to 30 μm.

**Figure 9:**
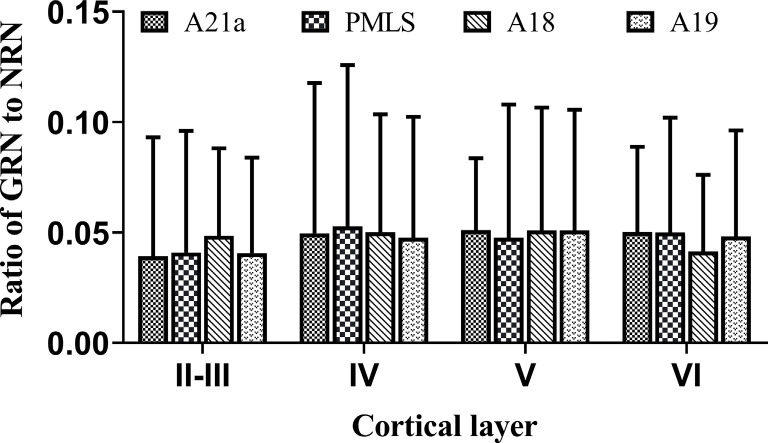
Histograms with error bars (SDs) represent the mean proportion of GABA/Retrobeads double-labeled neurons (GRNs) to NeuN/Retrobeads double-labeled neurons (NRNs) at cortical layer II–III, IV, V, and VI in cortical area 21a (A21a), PMLS, 18 (A18), and 19 (A19), respectively. The mean value was an average of counts from 30 neighboring sections (10 sections/cat).

The inhibitory GABA-positive neurons were found at all cortical layers in different cortical areas (Figure 8). However, red Retrobeads-traced neurons (RNs) in all layers exhibited very scarce overlapping with GABA-positive neurons (Figure 8). As a result, the number of GBA/Retrobeads double-positive neurons (GRN) was often counted as zero in many AOIs. Therefore, we compared the inter-area difference of GRN/NRN at different cortical layers using nonparametric tests with Kruskal–Wallis H. The results showed that the mean ratio ofGRN/NRN in cortical layer II–III, IV, V, and VI had no significant difference among different cortical areas (layer II–III: $\chi_{\left(3\right)}^{2}$ = 0.554, *p* = 0.907; layer IV: $\chi_{\left(3\right)}^{2}$ = 0.063, *p* = 0.996; layer V: $\chi_{\left(3\right)}^{2}$ = 0.71, *p* = 0.871; and layer VI: $\chi_{\left(3\right)}^{2}$ = 1.429, *p* = 0.699; Figure 9). Further, the mean ratio of GRN/NRN across all cortical areas showed no significant variation among cortical layer II–III (0.042 ± 0.048), IV (0.050 ± 0.062), V (0.050 ± 0.051), and VI (0.047 ± 0.043; $\chi_{\left(3\right)}^{2}$ = 1.213, *p* = 0.75); the mean ratio of GRN/NRN across all cortical layers had no significant difference among cortical area A21a (0.047 ± 0.049), PMLS (0.048 ± 0.060), A18 (0.048 ± 0.046), and A19 (0.047 ± 0.050; $\chi_{\left(3\right)}^{2}$ = 0.048, *p* = 0.997; Figure 9).

The comparisons above indicated that the mean ratio of GRN/NRN was very low at all cortical layers of all studied cortical areas, and showed no inter-area and inter-layer difference. Specifically, the mean ratio of GRN/NRN across all cortical layers in A21a (0.047 ± 0.049), PMLS area (0.048 ± 0.060), A18 (0.048 ± 0.046), and A19 (0.047 ± 0.050) was significantly lower than that of CRN/NRN (A21a: 0.82 ± 0.09; PMLS area: 0.80 ± 0.13; A18: 0.61 ± 0.12; A19: 0.62 ± 0.15; A21: *p* < 0.0001; PMLS area: *p* < 0.0001; A18: *p* < 0.0001; and A19: *p* < 0.0001; Figures 7, 9).

## Discussion

### Characteristics of Feedback Neurons in the High-Level Visual Cortex

It is traditionally assumed that visual information is processed in a feedforward hierarchical model that simple visual features are coded at the primary (V1) or low-level visual cortex, and complex visual attributes converged at higher-order visual areas for perceptual output (Juan and Walsh, 2003; Ro et al., 2003; Briggs and Usrey, 2011; Klink et al., 2017). Specifically, different visual representations are formed along segregated parallel pathways, with object shape or form information processed by the ventral stream from area V1→V3→V4 and motion/spatial location signatures processed by the dorsal stream from V1→V2→V5 (Lehky and Sereno, 2007; Brown, 2009; Kravitz et al., 2011; Mercier et al., 2017). Similar ventral and dorsal visual streams are also defined in the cat after the homolog area 17, 18, 19, 21a and PMLS are equated with V1, V2, V3, V4, and V5 based on electrophysiology evidence and area-specific behavioral observations (Dreher et al., 1993, 1996b; Payne, 1993; Wang et al., 2000, 2007; Shen et al., 2006; Tong et al., 2011; Connolly et al., 2012). Even though the importance of feedforward processing, evidence acquired in recent decades indicate that top-down influence from high-level visual or even nonvisual cortical areas can modulate neuronal response in the primary or low-level visual cortex (Wang et al., 2007; Thiele et al., 2009; Chen et al., 2014; Zhang et al., 2014; Huh et al., 2018), and thus plays critical roles during visual perceptual detection and perceptual learning (Alink et al., 2010; Miller et al., 2011; Al-Aidroos et al., 2012; Volberg et al., 2013; Morís Fernández et al., 2015). Nevertheless, the neuronal substrate carrying top-down influence to the V1 area is poorly understood. Based on neural circuit tracing techniques, some authors have taken great efforts to examine the corticocortical projections between low-level and higher-order visual cortex in the primate (Anderson and Martin, 2009), cat (Han et al., 2008; Connolly et al., 2012), ferret (Cantone et al., 2005; Khalil and Levitt, 2014) and mouse (Johnson and Burkhalter, 1994; Gonchar and Burkhalter, 2003; Laramée and Boire, 2014; Froudarakis et al., 2019). Complex back-projected connections are reported among varied cortical areas, such as V2 and V1 (Budd, 1998; Anderson and Martin, 2009), V5/V4/V3 and V1/V2 (Johnson and Burkhalter, 1997; Barone et al., 2000; Rockland and Knutson, 2000; Lyon and Kaas, 2002; Anderson and Martin, 2006) as well as area17, 18, 19, 21, PMLS and 7 (Symonds and Rosenquist, 1984; Shipp and Grant, 1991; Norita et al., 1996; Batardiere et al., 1998; Cantone et al., 2005; Han et al., 2008; Sherk, 2010). The distribution of tracer-labeled feedback neurons reported by different authors varied considerably with cortical layers (Symonds and Rosenquist, 1984; Budd, 1998; Rockland and Knutson, 2000; Cantone et al., 2005; Anderson and Martin, 2009) and cortical areas (Shipp and Grant, 1991; Batardiere et al., 1998; Anderson and Martin, 2006; Sherk, 2010). Factors leading to these variations are unclear. Recent imaging studies show that top-down influence affects the neural activity of the V1 area in a retinotopically specific manner (Griffis et al., 2015b, 2017), which raises the expectation that feedback projections may vary accordingly (Griffis et al., 2015a). Considering most previous studies perform a single or two tracer injection at the target cortical area in each animal (Symonds and Rosenquist, 1984; Batardiere et al., 1998; Barone et al., 2000; Rockland and Knutson, 2000; Sherk, 2010; Khalil and Levitt, 2014), it is probably that difference in the tracer injection site, depth and spreading range could have, at least partially, contributed to the variations in the number of tracer-labeled neurons. Further studies are needed to clarify this issue by comparatively examining feedback neurons after injection of tracers at different retinotopic coordinates in the V1 area.

The current study performed multiple tracer injections at a wide range of retinotopic locations (about 0–20° from the vertical and horizontal meridian, see also Supplementary Figure 1) in the V1 area (area 17) of each cat and released tracers at varied cortical depth from 200 to 2,000 μm (see section “Animal Preparation and Injection of Retrograde Tracer”). We found that tracer-labeled feedback neurons in the high-level visual cortex distributed widely in cortical layer II–III, IV, V, and VI except for layer I. Our statistical results showed that the higher-level visual cortical area 21a at the ventral stream of pathway and area PMLS at the dorsal stream had a comparable proportion of feedback neurons that back-projected directly to the V1 area, and the distribution of feedback neurons in these two higher-level visual areas was similar at cortical layer II–III, IV, V, and VI. These results suggest that the ventral and dorsal visual streams may closely interact through the V1 area during information processing, which is consistent with previous studies (Shen et al., 2006; Gilaie-Dotan et al., 2013; Zachariou et al., 2014; Huang et al., 2017; Mercier et al., 2017) and argues against the proposition of ventral vs. dorsal pathway segregation (Brown, 2009; Bracci and Op de Beeck, 2016; Milner, 2017). Further, this study also quantitatively compared the proportion of feedback neurons among different high-level visual cortical areas. Surprisingly, the result indicated that the mean proportion of feedback-to-V1 neurons at cortical layer II–III, IV, V, and VI in the higher-order visual area 21a and PMLS was significantly higher than in the intermediate visual area 18 and 19. This result suggests that information in the higher-order visual cortex may need to return to the V1 area for further strengthening or integration before proceeding to perceptual output. Our results may challenge the traditional feedforward hierarchical processing model (Silvanto, 2014, 2015) and support the reverse hierarchy theory or interactive model proposing that recurrent connections between V1 and higher-order visual areas form functional circuits mediating aware and unaware visual perception (Johnson and Burkhalter, 1997; Juan and Walsh, 2003; Tong, 2003; Juan et al., 2004; Silvanto et al., 2005; Koivisto et al., 2010; Froudarakis et al., 2019).

### Mechanisms of Top-Down Influence

Although it is widely aware of the importance of top-down influence in visual perception and learning (Alink et al., 2010; Miller et al., 2011; Al-Aidroos et al., 2012; Volberg et al., 2013; Morís Fernández et al., 2015). The underlying brain mechanisms remain elusive. An increasing number of studies show that high-level visual and even nonvisual cortical areas may affect neuronal responses and thus modulate visual information encoding in the primary or low-level visual cortex, such as stimulus selectivity and contrast sensitivity (Wang et al., 2007; Thiele et al., 2009; Chen et al., 2014; Zhang et al., 2014; Huh et al., 2018). However, results reported by different research groups are diverse or even opposite. For example, some authors find out that top-down influence can facilitate the responses of V1 neurons and thus enhance their orientation or direction selectivity (Wang et al., 2000, 2007, 2010; Galuske et al., 2002; Huang et al., 2007; Tong et al., 2011; Moldakarimov et al., 2014; Zhang et al., 2014; Nurminen et al., 2018; Keller et al., 2020), whereas others report that top-down influence is suppressive and lowers neuronal response in the low-level visual cortex (Murray et al., 2002; Alink et al., 2010; Nassi et al., 2013; Klein et al., 2014; Hishida et al., 2019; Maniglia et al., 2019; Ranson et al., 2019). A critical way to reconcile these contradictions is to determine the cell type of feedback neurons as well as the neurotransmitter system used by the feedback circuitry. Nevertheless, limited information has been provided by previous studies although a few authors have shed some light on this issue (Gonchar and Burkhalter, 2003; Zhang et al., 2014; van Loon et al., 2015; D’Souza et al., 2016; Nurminen et al., 2018). In this study, we classified feedback neurons using the fluorescent double-labeling method after neuronal tracing and brain tissue sectioning. Our results showed that most tracer-labeled feedback neurons in the higher-order visual cortical area 21a and PMLS (about 75–86%) and more than half feedback neurons in the intermediate visual area 18 and 19 (about 54–67%) are CaMKII-positive excitatory neurons, whereas very few feedback neurons are identified as inhibitory GABAergic ones (around 5%). This result is consistent with the proposition that top-down influence may use primarily excitatory amino acid neurotransmitters (Johnson and Burkhalter, 1994; Liang et al., 2007; Anderson and Martin, 2009; van Loon et al., 2015; Han and VanRullen, 2016), and support feedback facilitation effects reported previously (Wang et al., 2000, 2007, 2010; Chen et al., 2014; Pafundo et al., 2016; Keller et al., 2020). Interestingly, the total percentage of CaMKII-positive plus GABA-positive feedback neurons measured in this study was lower than 100%, especially in the intermediate visual area 18 and 19. The reasons could be partially related to the counting standard we set for feedback neurons with ≥75% overlapping with NeuN-, CaMKII- and GABA-positive neurons. The amounts of different types of neurons could be underestimated. An additional reason was likely that a part of feedback neurons might use other neurotransmitters that were not visualized in the current study, such as noradrenergic, cholinergic, and serotonin neurotransmitter systems (Hirata et al., 2006; Challis and Berton, 2015; Datta et al., 2019). Further studies are needed to clarify these possibilities.

Even though a dominant drive of excitatory feedback can increase neuronal responses in the V1 or low-level visual areas, we cannot exclude the involvement of other neurotransmitter systems, such as GABAergic inhibition (Zhang et al., 2014; Mazo et al., 2016), because the different source of feedback influences may differently activate recurrent neuronal circuits and modulate the balance between excitation and inhibition in the V1 area (Johnson and Burkhalter, 1997; Schwabe et al., 2006; Yang et al., 2013; D’Souza et al., 2016; Kamiyama et al., 2016; Liang et al., 2017). This may explain why some authors observe bidirectional effects of both enhancement and suppression in the V1 or low-level visual cortex after modification of top-down influence (Gazzaley et al., 2005; Johnson and Johnson, 2009; Cox et al., 2019). Additionally, feedback-derived disinhibition may also occur in the V1 area (Zhang et al., 2014; Tremblay et al., 2016; Feldmeyer et al., 2018) although the feedback neurons are mostly excitatory as shown in this study. Further studies are needed to examine the dynamics of neurotransmitter systems in the V1 or low-level visual areas during top-down influence manipulation to elucidate the underlying neuronal and molecular mechanisms.

## Data Availability Statement

The original contributions presented in the study are included in the article/Supplementary Materials, further inquiries can be directed to the corresponding author.

## Ethics Statement

The animal study was reviewed and approved by the Academic and Ethics Committee of Anhui Normal University.

## Author Contributions

HP and TH: design of the work. HP, SZ, DP, HY, JD, ZY, QW, and QS: animal preparation, heart perfusion, brain tissue sectioning, fluorescent double-labeling, and data statistics. All authors have a contribution to data interpretation, manuscript drafting and revision. All individuals designated as authors qualify for authorship, and all those who qualify for authorship are listed. All authors have approved the final version of the manuscript and agree to be accountable for all aspects of the work in ensuring that questions related to the accuracy or integrity of any part of the work are appropriately investigated and resolved. All authors contributed to the article and approved the submitted version.

## Conflict of Interest

The authors declare that the research was conducted in the absence of any commercial or financial relationships that could be construed as a potential conflict of interest.

## Supplementary Material

SUPPLEMENTARY FIGURE 1 Brain tissue image showing the injection site of red Retrobeads in area 17 (A17). The solid black dot in A17 is the estimated site of retinotopic area centralis at the intersection of vertical and horizontal meridian according to previous studies (Tusa et al., 1978, 1979; Connolly et al., 2012). The six dashed line circles along the anterior (A)-to-posterior (P) direction in A17 represent injection location at Horsley-Clarke coordinates of P1/L1.5, P2/L2, P3/L2.5, P4/L3, P5/L3.5, and P6/L3.5, which correspond to the retinotopic coordinates within approximately 0–20° from the vertical and horizontal meridian (Tusa et al., 1978, 1979; Connolly et al., 2012). A18, A19, A21a, A7, and PMLS represent the visual cortical area 18, 19, 21a, 7, and PMLS, respectively. The dashed lines are estimates of landmarks between different visual areas. The scale bar equals to 1.0 mm.
