# Supplementary figures and images for: Characterization of Feedback Neurons in the High-Level Visual Cortical Areas That Project Directly to the Primary Visual Cortex in the Cat
Source: Front Neuroanat. 2021 Jan 8;14:616465. doi: 10.3389/fnana.2020.616465 (PMC7820340; doi:10.3389/fnana.2020.616465)

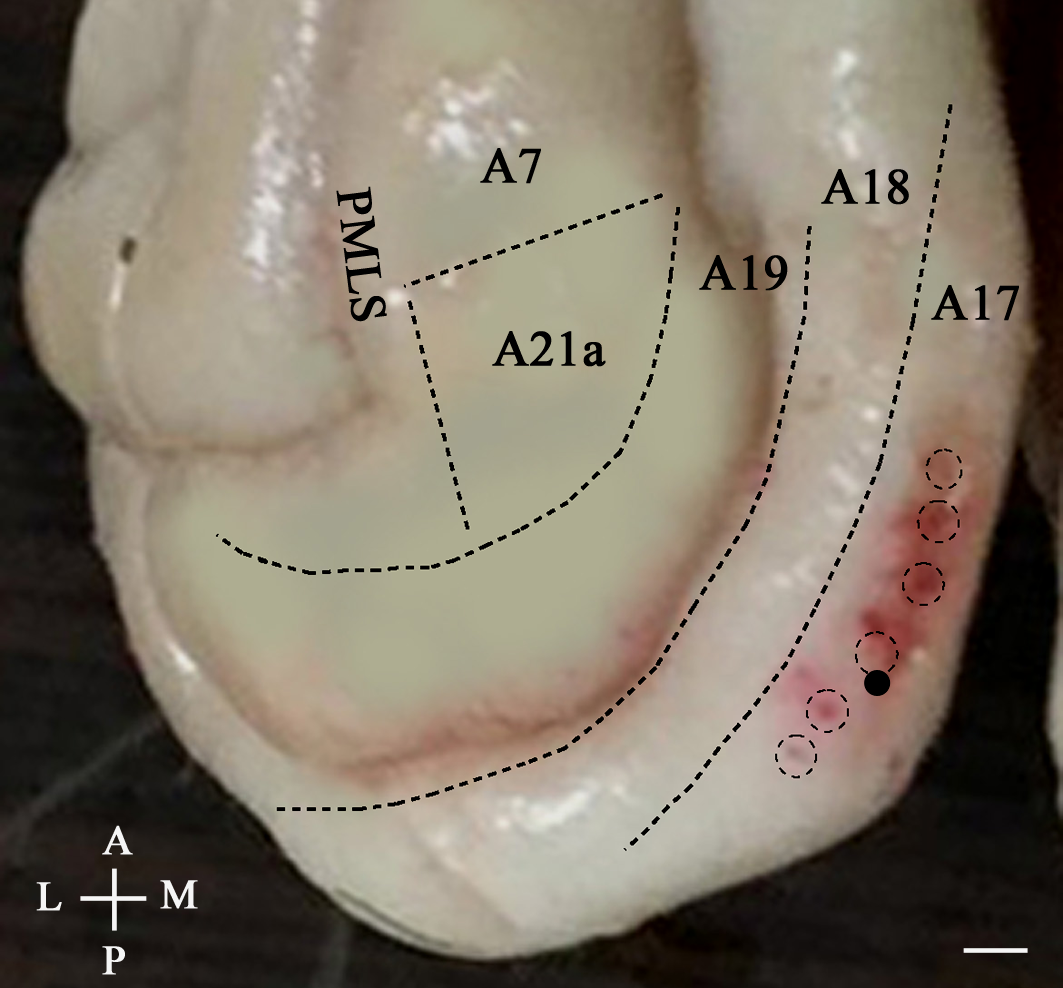

Supplement: SUPPLEMENTARY FIGURE 1 — Brain tissue image showing the injection site of red Retrobeads in area 17 (A17). The solid black dot in A17 is the estimated site of retinotopic area centralis at the intersection of vertical and horizontal meridian according to previous studies (Tusa et al., 1978, 1979; Connolly et al., 2012). The six dashed line circles along the anterior (A)-to-posterior (P) direction in A17 represent injection location at Horsley-Clarke coordinates of P1/L1.5, P2/L2, P3/L2.5, P4/L3, P5/L3.5, and P6/L3.5, which correspond to the retinotopic coordinates within approximately 0–20° from the vertical and horizontal meridian (Tusa et al., 1978, 1979; Connolly et al., 2012). A18, A19, A21a, A7, and PMLS represent the visual cortical area 18, 19, 21a, 7, and PMLS, respectively. The dashed lines are estimates of landmarks between different visual areas. The scale bar equals to 1.0 mm. [file Image_1.TIF]
